# Antegrade Posterior Column Acetabulum Fracture Screw Fixation via Posterior Approach: A Biomechanical Comparative Study

**DOI:** 10.3390/medicina59071214

**Published:** 2023-06-28

**Authors:** Till Berk, Ivan Zderic, Peter Schwarzenberg, Ludmil Drenchev, Hristo Kostov Skulev, Roman Pfeifer, Tatjana Pastor, Geoff Richards, Boyko Gueorguiev, Hans-Christoph Pape

**Affiliations:** 1 AO Research Institute Davos, 7270 Davos, Switzerland; ivan.zderic@aofoundation.org (I.Z.); peter.schwarzenberg@aofoundation.org (P.S.); tatjana.pastor@aofoundation.org (T.P.); geoff.richards@aofoundation.org (G.R.); boyko.gueorguiev@aofoundation.org (B.G.); 2 Department of Trauma, University Hospital Zurich, 8091 Zurich, Switzerland; roman.pfeifer@usz.ch (R.P.); hans-christoph.pape@usz.ch (H.-C.P.); 3 Harald-Tscherne Laboratory for Orthopedic and Trauma Research, University of Zurich, 8091 Zurich, Switzerland; 4 Institute of Metal Science ‘‘Acad. A. Balevski’’, Bulgarian Academy of Sciences, 1574 Sofia, Bulgaria; ljudmil.d@ims.bas.bg (L.D.); skulev@ims.bas.bg (H.K.S.); 5 Department of Plastic and Hand Surgery, Inselspital University Hospital Bern, University of Bern, 3012 Bern, Switzerland

**Keywords:** acetabulum fracture, antegrade screw fixation, biomechanics, cannulated compression headless screw, feasibility, posterior approach, posterior column fracture

## Abstract

*Background and Objectives*: Minimally invasive surgeries for acetabulum fracture fixation are gaining popularity due to their known advantages versus open reduction and internal fixation. Antegrade or retrograde screw fixation along the long axis of the posterior column of the acetabulum is increasingly applied in surgical practice. While there is sufficient justification in the literature for the application of the anterior approach, there is a deficit of reports related to the posterior approach. The aim of this study was to evaluate the biomechanical competence of posterior column acetabulum fracture fixation through antegrade screw placement using either a standard cannulated screw or a cannulated compression headless screw (CCHS) via posterior approach. *Materials and Methods*: Eight composite pelvises were used, and a posterior column acetabulum fracture according to the Letournel Classification was simulated on both their left and right sides via an osteotomy. The sixteen hemi-pelvic specimens were assigned to two groups (*n* = 8) for either posterior column standard screw (group PCSS) or posterior column CCHS (group PCCH) fixation. Biomechanical testing was performed by applying steadily increased cyclic load until failure. Interfragmentary movements were investigated by means of motion tracking. *Results*: Initial stiffness demonstrated significantly higher values in PCCH (163.1 ± 14.9 N/mm) versus PCSS (133.1 ± 27.5 N/mm), *p =* 0.024. Similarly, cycles and load at failure were significantly higher in PCCH (7176.7 ± 2057.0 and 917.7 ± 205.7 N) versus PCSS (3661.8 ± 1664.5 and 566.2 ± 166.5 N), *p* = 0.002. *Conclusion*: From a biomechanical perspective, CCHS fixation demonstrates superior stability and could be a valuable alternative option to the standard cannulated screw fixation of posterior column acetabulum fractures, thus increasing the confidence in postoperative full weight bearing for both the patient and treating surgeon. Whether uneventful immediate postoperative full weight bearing can be achieved with CCHS fixation should primarily be investigated in further human cadaveric studies with a larger sample size.

## 1. Introduction

Minimally invasive surgeries for acetabulum fracture fixation are persistently gaining popularity due to their well-known advantages versus open reduction and internal fixation [1,2,3]. Antegrade or retrograde lag screw fixation along the long axis of the posterior column of the acetabulum is increasingly used in surgical practice to treat transverse or T-type acetabulum fractures [4]. The antegrade screw placement can be achieved via an anterior or posterior approach and is still a relatively new fixation method [5]. The anterior approach for antegrade screw placement is attained via the ilioinguinal approach utilizing a reduced lateral window [4]. On the other hand, the posterior approach can be accomplished through a minimally invasive incision at the posterior portion of the iliac crest [6]. The anterior approach for antegrade screw placement has already been sufficiently explored in several studies using cadavers, computer models, and in vivo experiments [3,5,7,8,9]. In addition to the ideal entry point, the narrow channel for screw placement was specifically addressed in these investigations. Furthermore, a variety of studies focusing on retrograde fixation of the posterior column have been performed [10,11,12,13]. However, there is a deficit of reports in the literature related to the posterior approach for antegrade lag screw placement. In cases with non-displaced or only minimally displaced posterior column fractures treated via antegrade screw fixation, early mobilization of the patients to avoid complications from immobilization are promoted [6,8]. Nevertheless, the available literature still seems to be too limited to draw any substantiated conclusions. Therefore, the aim of this study was to evaluate the biomechanical competence of posterior column acetabulum fracture (PCF) fixation through antegrade screw placement using either a standard cannulated screw or a cannulated compression headless screw (CCHS) via posterior approach. A further aim was to assess whether this approach provides sufficient stability of fixation for consideration of postoperative full weight bearing.

## 2. Materials and Methods

Eight composite pelvises were used (Model 4060^®^, Synbone AG, Zizers, Switzerland). Their right and left sides were separated, and each one of them was considered an independent specimen for testing. A PCF was set in line with the Letournel Classification via osteotomizing [14]. A custom-made template was used for the standardization of all osteotomies. The sixteen hemi-pelvic constructs were assigned to two groups of eight specimens each (*n* = 8) and an equal number of right and left sides for implantation. In group ‘‘posterior-column-standard-screw-fixation’’ (PCSS), fracture treatment was performed using a single partially threaded 6.5 mm cannulated screw made of stainless steel (316LVM), 90 mm in length. In group ‘‘posterior-column-CCHS-fixation’’ (PCCH), fracture treatment was performed using a single partially threaded 6.5 mm CCHS made of titanium alloy (Ti-6Al-4V ELI), 90 mm in length. All implants were produced by the same manufacturer (DePuy Synthes, Zuchwil, Switzerland).

**Figure 1:**
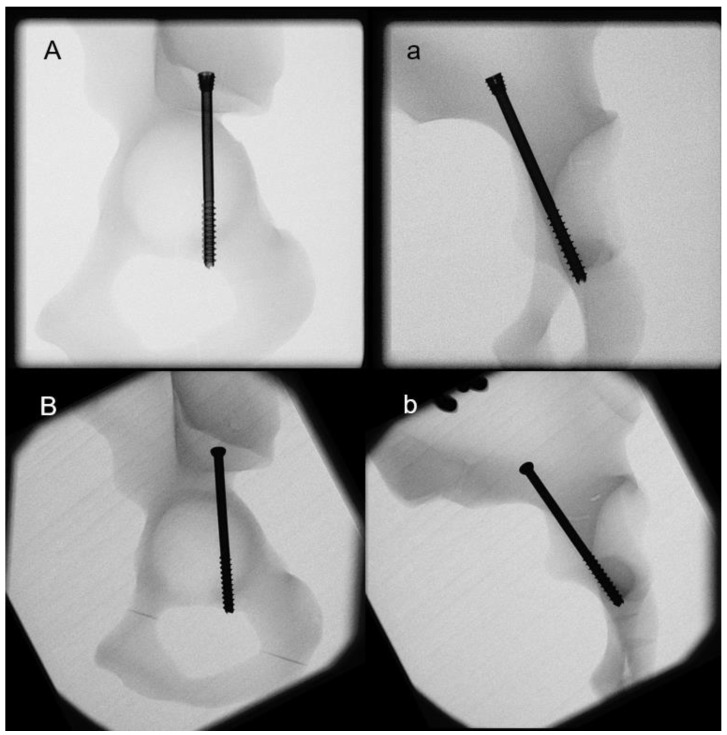
X-rays after implantation of specimens from groups PCCH (**A**,**a**) and PCSS (**B**,**b**).

After PCF reduction, a 2.8 mm Kirschner (K-) guide wire was placed in the acetabulum across the osteotomy line and cephalad through the proximal part of the superior pubic ramus. Its entry point was determined radiologically at the posterior aspect of the iliac crest. Fluoroscopic monitoring was performed to avoid any perforations or cortical disruptions during the wire placement. After pilot drilling, the insertion of each cannulated screw proceeded along the guide wire, with tightening in accordance with the operating surgeon’s best practice. All fixation procedures followed the surgical guidelines of the implant producer. After instrumentation, anterior-posterior, obturator oblique, inlet, and iliac oblique X-rays were performed for documentation and verification (Figure 1). One marker set was attached to each specimen’s fragment for motion tracking.

### 2.1. Biomechanical Testing

**Figure 2:**
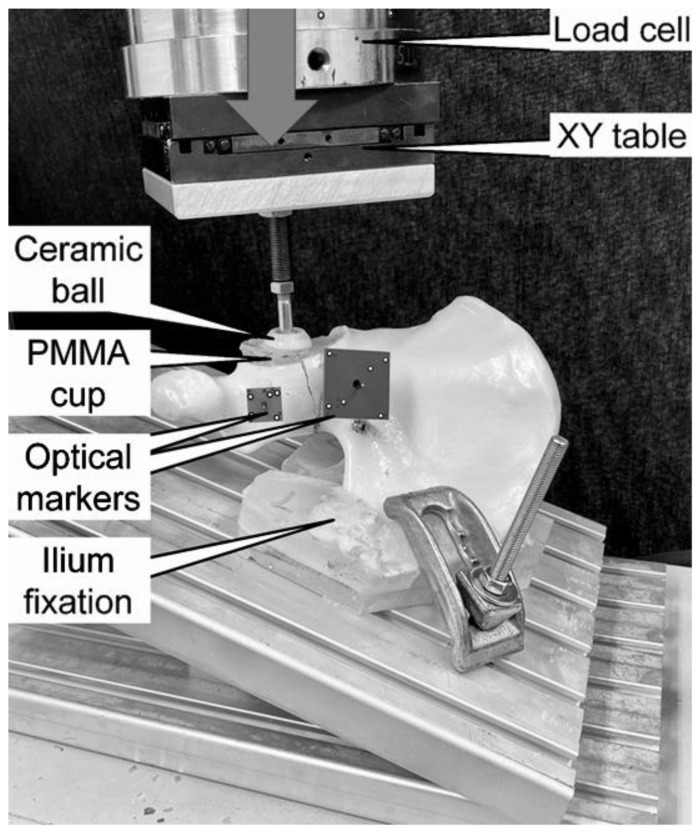
Setup with a specimen mounted for biomechanical testing. The vertical arrow denotes the loading direction.

Biomechanical testing was run on a material test system (Mini Bionix II 858; MTS Systems, Eden Prairie, MN, USA) equipped with a 4 kN load cell (HUPPERT 6, HUPPERT GmbH, Herrenberg, Germany). The setup was implemented from previous studies (Figure 2) [15]. Each hemi-pelvic specimen was tested in an inverted, upright standing position, resting on a base plate inclined at 20° to the machine base in the frontal plane. Both the symphyseal medial aspect and the sacroiliac joint were positioned flush with the base plate in accordance with Morosato et al. [16]. The sacroiliac joint was additionally fixated to the base plate using two polymethylmethacrylate (PMMA, SCS-Beracryl D-28, Suter Kunststoffe AG/Swiss-Composite, Fraubrunnen, Switzerland) blocks allowing standardized fixation of all specimens.

The compression along the machine axis was applied to the acetabulum by means of a ball of 28 mm radius. A molded PMMA hemi-spherical cavity was inserted in the acetabulum for homogenous load transfer. This configuration targeted a simulation of a hip joint reaction force trajectory during walking, as reported by Bergmann et al. [17].

The test protocol considered an initial non-destructive quasi-static ramped loading in axial compression (20–200 N, rate 18 N/s), followed by steadily increasing cyclic loading at a rate of 2 Hz, featuring a physiological profile of each cycle [17]. While the valley load was kept constant at 20 N, the initial peak load of 200 N was steadily increased by 0.1 N/cycle until 10 mm displacement of the machine actuator relative to the test start. This stop criterion proved to be adequate for destructive testing with the catastrophic failure of all specimens [18,19].

### 2.2. Data Collection and Analysis

Uniaxial machine data in terms of displacement and load were collected at 200 Hz. Based on this, construct stiffness was derived from the load–displacement curve of the quasi-static ramp within the linear range of 100 to 160 N.

The markers’ coordinates were collected during testing at 20 Hz (Aramis SRX, Carl Zeiss GOM Metrology GmbH, Braunschweig, Germany) to calculate the relative movements between the two fragments of each specimen. Based on this, three parameters were analyzed between 1000 and 6000 test cycles at time intervals of 1000 cycles under peak loading with respect to the cyclic test start: (1) fracture displacement, i.e., the translational interfragmentary displacement at the most anterior aspect of the acetabulum fracture portion; (2) fracture gap opening, i.e., the out-of-osteotomy-plane angular interfragmentary displacement; and (3) fracture gap twisting, i.e., the within-fracture-plane angular interfragmentary displacement. It is worth noting that 6000 was the highest rounded number of cycles without indicated failure of any specimen.

A fracture displacement of 1 mm was considered to set a clinically relevant criterion for failure. The number of cycles until its fulfillment under peak loading—i.e., cycles to clinical failure—was evaluated along with the corresponding peak load—i.e., clinical failure load. In addition, the number of cycles until catastrophic specimen failure—i.e., cycles to catastrophic failure—was evaluated along with the corresponding catastrophic failure load and fracture displacement at catastrophic failure.

Statistical evaluation was done using SPSS software (Version 27, IBM SPSS, Armonk, NY, USA). The normality of data distribution was proved with Shapiro–Wilk test. Construct stiffness, cycles to clinical and catastrophic failure, clinical and catastrophic failure load, and fracture displacement at catastrophic failure were statistically compared between the groups with Independent-Samples T-test. Fracture displacement, fracture gap opening, and fracture twisting between 1000 and 6000 test cycles at time intervals of 1000 cycles were statistically compared between the groups via General Linear Model Repeated Measures and Bonferroni posthoc tests for multiple comparisons. An overall significance level of 0.05 was considered.

## 3. Results

**Figure 3:**
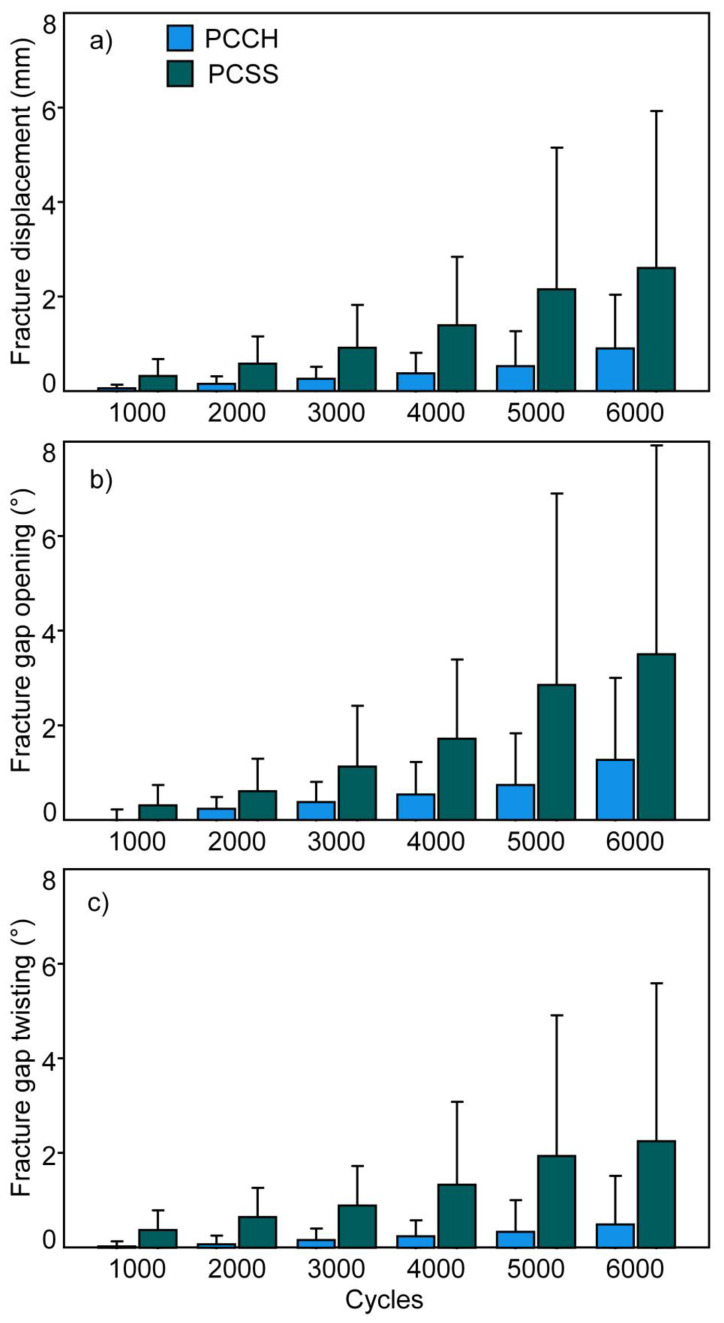
Fracture displacement (**a**), fracture gap opening (**b**), and fracture gap twisting (**c**), presented for each separate group (PCCH and PCSS) over the time points between 1000 and 6000 test cycles at time intervals of 1000 cycles in terms of mean value and standard deviation.

Construct stiffness was significantly higher in PCCH (163.1 ± 14.9 N/mm (mean ± standard deviation)) versus PCSS (133.1 ± 27.5 N/mm), *p =* 0.024. The values of the parameters analyzed between 1000 and 6000 test cycles at time intervals of 1000 cycles are summarized in Figure 3. Each of them was associated with significantly higher values detected for PCCH versus PCSS, *p* ≤ 0.006.

**Figure 4:**
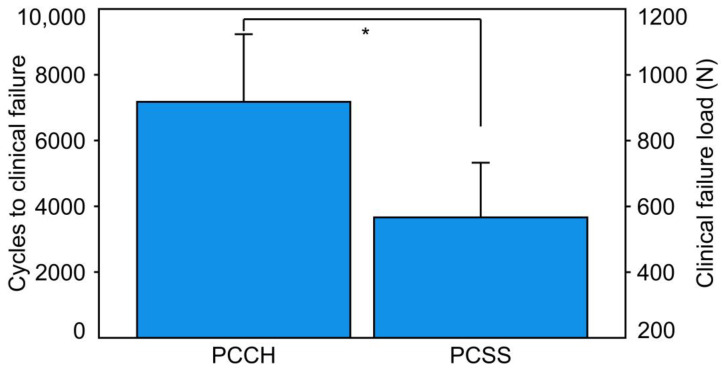
Cycles to clinical failure and corresponding clinical failure load presented for each separate group (PCCH and PCSS) in terms of mean value and standard deviation. The star indicates a significant difference.

Cycles to clinical failure and clinical failure load were significantly higher in PCCH (7176.7 ± 2057.0 and 917.7 ± 205.7 N) versus PCSS (3661.8 ± 1664.5 and 566.2 ± 166.5 N), *p =* 0.002 (Figure 4). Cycles to catastrophic failure and catastrophic failure load were higher in PCCH (7782 ± 1610 and 978.2 ± 161.0 N) versus PCSS (7235 ± 1100 and 923.5 ± 110.0 N), without significant differences between them, *p* = 0.441. Fracture displacement at catastrophic failure was significantly less in PCCH (1.29 ± 0.49 mm) versus PCSS (3.03 ± 1.69 mm), *p* = 0.023. The catastrophic failure modes of the specimens were characterized by either fracture displacement due to a pullout of the fixation screw or by a supraacetabular horizontal fracture.

## 4. Discussion

This study investigated the biomechanical competence of PCF treated with either a standard 6.5 mm cannulated screw or a 6.5 mm CCHS designed for safe antegrade placement in the posterior acetabulum column via posterior approach to identify that:

1. PCCH was associated with significantly higher construct stiffness versus PCSS.
2. PCCH demonstrated significantly fewer interfragmentary movements during the first 6000 test cycles compared to PCSS.
3. Cycles to clinical failure and clinical failure load were significantly higher for PCCH versus PCSS.

The existing evidence-based work related to the postoperative management of unstable acetabulum fractures is scant [20]. In addition, some biomechanical studies questioned the justification of restrictive weight bearing in such cases [17,21]. Although both constructs in the present study failed catastrophically at a statistically comparable load level, the CCHS fixation demonstrated significantly (1) less interfragmentary movements, (2) higher construct stiffness, (3) higher number of cycles until clinically relevant failure, (4) higher clinical failure load, and (5) less fracture displacement at catastrophic failure, when compared to one of today’s standard screw treatments of PCFs. These findings could provide further arguments in favor of immediate postoperative full weight bearing following surgically treated posterior column fractures.

A clinical study on geriatric patients with minimally displaced or non-displaced acetabulum fractures (T-type, transverse, or column fractures) investigated fixation with two retrograde percutaneously placed 7.3 mm cannulated cancellous screws and a full weight bearing scenario initiated 4 weeks after the operation. The authors reported neither intraoperative nor postoperative complications, secondary fragment displacement, and screw failures [12].

Both 6.5 mm and 7.3 mm cannulated screws implement the standard diameters for screw fixation of posterior column fractures. The implants used in the two groups of the current study had the same diameter of 6.5 mm; however, apart from their non-identical design, they were made of different materials, namely stainless steel and titanium alloy. One could argue that these different characteristics could explain the considerable differences detected between the fixation techniques. However, no breakage of any screw type occurred during testing. Rather, the artificial bones failed mechanically, or a fracture displacement was observed. In view of the results, the authors believe that the influence of the implant material can therefore be primarily neglected during the interpretation of the data, comprehending that this discussion point cannot be addressed conclusively in the current study. Additionally, literature work concludes that steel and titanium screws provide comparable stability [22,23,24].

Regarding the selected implant size, fixation with a larger screw could result in better stability. However, it must be considered that the anatomical bone tract, where the antegrade screw is placed, is exceptionally narrow. A thicker screw could therefore result in a more difficult surgery and possibly provoke iatrogenic damage.

A study analyzing the dimensions of the pelvis and acetabulum reported that the average maximum diameter of the posterior column was 11.40 mm (ranging from 9.40 to 13.30 mm) [25]. A similar work focusing on retrograde screw placement pointed out that the diameters of the posterior column corridor were 17.21 ± 1.41 mm in males and 15.54 ± 1.51 mm in females, therefore being theoretically sufficient to accommodate three 7.3 mm screws in males and three 6.5 mm screws in females [2].

These previous findings are indirectly in agreement with the results of the current study. No perforations of the acetabulum or the surrounding structures were observed using either the standard cannulated screw or the CCHS. Furthermore, no prominent occurrence of screws was observed in either group, neither under radiographic fluoroscopy nor under direct visualization. Therefore, both screw diameters appear to be safe for antegrade fixations through the posterior window. The length of both screw types was chosen to be 90 mm, such that the tip of the screws would just perforate the distal cortex of the proximal inferior pubic ramus.

A cadaveric study suggested that the insertion of a 6.5 mm lag screw with an average length of 104.8 ± 4.2 mm is feasible in the posterior column via the anterior approach with no unintentional extraosseous or intraarticular screw prominence within the named safe zone [8]. A different computational study addressing the same research question concluded that the safe zone was sufficiently large for placement of multiple screws through the anterior window, including 7.0 mm screws [3].

A biomechanical investigation comparing antegrade screw fixation via the anterior approach versus standard plating of transverse acetabulum fractures concluded that the screw technique provided higher fixation strength compared to plate fixation [26].

The posterior window for antegrade screw fixation of posterior column fractures allows screw insertion almost perpendicular to the fracture plane. This, together with a good fracture compression, should provide ideal conditions for anatomical reduction of the fracture. Due to the supplementary proximal thread in the CCHS design, additional anchoring in the near cortex is achieved. This could lead to higher compression forces applied to the fracture, or the screws could possibly be tightened more firmly. A washer, added to the standard screw, could achieve a comparable effect; however, its use is not recommended in this region [27]. The structural CCHS characteristics could also provide an explanation of the current results. Their investigation would require torque and fracture compression measurements that were out of the focus of this study.

When comparing the anterior versus posterior approach for antegrade screw placement, the posterior approach appears to be a much more minimally invasive variant. For the posterior entry point, only a stab incision and blunt dissection through the musculature down to the bone would be indicated. In addition, an entirely percutaneous approach is also very conceivable. In contrast, more extensive preparation and a significantly larger incision are considered in relation to the anterior approach [4]. Furthermore, the necessary surgical time and blood loss are likely to be significantly less when using the posterior versus anterior approach, depending on the operator.

### Strength and Limitations

The use of a composite bone model represents the main limitation of the present work. Nevertheless, the latter features an investigation with the use of an innovative implant and a focus on an understudied topic with very limited data available in the literature. A human cadaveric study, performed as a next step after gaining knowledge from the current one, would be more ethically justifiable. It is well-known that composite pelvic specimens allow for cost-effective standardized examinations to overpower the larger variations in bone quality and shape of human cadaveric bones [28,29,30,31]. Further, artificial bone specimens have been used in various biomechanical studies, including pelvic investigations [28,32,33,34,35]. Moreover, the restricted availability of cadavers affects the sample size of biomechanical studies while the use of artificial bones minimizes the confounding variability of test results [34,36]. The two types of screws used in the current study were manufactured from different materials that could have influenced the results. However, no screw breakage occurred in any of the specimens during biomechanical testing. Therefore, in our opinion, the material of the screws had a primarily negligible influence on the study results. Finally, the arbitrary set criterion for clinically relevant specimen’s failure worked well in the current biomechanical environment, because in most of the tests the specimens revealed an abrupt drop in stability over time in close relation to it.

## 5. Conclusions

From a biomechanical perspective, CCHS fixation demonstrates superior stability and could be a valuable alternative option to the standard cannulated screw fixation of posterior column acetabulum fractures, thus increasing the confidence in postoperative full weight bearing for both the patient and treating surgeon. Whether uneventful immediate postoperative full weight bearing can be achieved with CCHS fixation should primarily be investigated in further human cadaveric studies with a larger sample size.

## Data Availability

The datasets analyzed during the current study are available from the corresponding author upon reasonable request.
